# Early Viability of the Fœtus

**Published:** 1866-09

**Authors:** Wm. Kennedy

**Affiliations:** New Orleans


*Early Viability of the Fœtus—an Extraordinary Case.* Reported by Wm. Kennedy, M. D., New Orleans.

In 1845, Mrs. A. B., primipara, suffered, as she thought, during one whole night with colic. I saw her next morning, when I recognized that she was in labor, which had progressed so far that I made no attempt to arrest it. Within a half hour after my arrival she gave birth to a fœtus. It was not more than eight inches long, and was as red as a piece of raw beef. True dermoid tissue could not be said to be organized, its general investiture being so delicate a membrane, as it were, that the eye could look through it on the tissues beneath. The eyes were still closed; there was no trace of cilia or supercilia; its chest was about two inches broad; the arms and legs were very slender, and the toes and fingers devoid of any traces of nails. The head was about the size of a small orange. The respiration was so feeble as scarcely to be perecptible, and not a sound was uttered after birth. I was almost afraid to handle it, as I could not divest myself of the idea that the slightest pressure of the fingers would thrust them into the soft, red, jelly-like mass before me. When I raised it from the couch, and laid it in the length of my left hand, the head lay on the convexity of my flexed fingers, the chest and breech in the palm, and the feet reached almost an inch beyond the wrist.

I wrapped it carefully in batting, and carefully attended to maintaining a proper surrounding temperature. It was fed drop by drop with sugar and water every four or five minutes, and later, when the mother could supply it with milk, half a teaspoonful was given every half hour or hour. Within three weeks after birth it had a mild attack of trismus. During treatment I gave it frequent baths in a tumbler. The period of infancy was one of the most stormy I ever saw. Hydrocephalus, cholera-infantum, measles, diarrhœa, are some of the many affections it suffered from during that time, and up to three or four years. When last I saw him he was a fine healthy boy of twelve years, and gave promise of a vigorous manhood.

I should have been pleased to have furnished a more accurate account of this case. The importance of the subject demands it. But my note-book, carefully preserved during many years of arduous practice, and which I hoped, in my declining days, to make useful to my brethren, through the medium of the press, has, like my library and every vestige of household material, passed from my possession under the ruthless hand of destructive war.

---

The report of this case is a matter of the highest importance, both in a humanitarian and medico-legal point of view; and that which gives additional value to the report is its entire reliability. No man stands higher in his profession in this community than Dr. Kennedy, and his observations may be relied on as strictly accurate. It is certainly a matter for real regret, that Dr. K. should have lost his complete notes of the case, as it would have been most truly interesting to have watched the progress of development, from week to week, of so young a fœtus thrown into the external world. We consider this case as the most important one on record, when we consider the reliability of the author of the report, the exceeding youth of the fœtus, the very limited development, the stormy history of its infantile life, and the successful issue. In a humanitarian point of view, it must interest the scientific obstetrician, as it must force on him a sense of the imperative duty of refraining from unnecessary destruction of fœtal life, even at so early a period; as well as the duty of carefully attending to every fœtus born with evidences of vitality under his hands. We have an abiding conviction, that not a few fœtal lives are sacrificed in every community through neglect of the indications thus made manifest.

Again, in a medico-legal point of view, if the fact of viability, and consequent claim to inheritance of titles or estates, shall have always heretofore been denied the fœtus of less than six months, this case would seem to set aside all prejudice on the subject. The law, in such cases, can justly take cognizance only of authentic facts, and cannot arbitrarily fix a certain age of viability. In Wharton and Stille's Medical Jurisprudence, we find accounts of a fœtus born on the one hundred and seventy-ninth day of gestation, living to the age of four months, and then dying of epidemic disease; of another, born on the one hundred and fifty-eighth day of gestation, weighing one pound, measuring eleven inches in length, and being still alive three years after; and another born at twenty-seven weeks and living still eleven years after; and another, thought to be not more than five months, as, three weeks after birth, it weighed only one pound thirteen ounces, measuring between eleven and thirteen inches, and surviving its birth one year and nine months.

Dr. Kennedy's case takes precedence of all these, as the description of its length and general development go to indicate clearly that it could not have been more than one hundred and forty to one hundred and forty-five days old at birth.

The Napoleonic Code would seem to fix legal viability at one hundred and forty-eight days, but with the light of Dr. Kennedy's case before us, should the reputation of a woman, or the hereditary rights of her child, be compromised under the force of such arbitrary dicta? Or will a husband be justified in sus pecting the integrity of his wife, or in seeking divorce from her, on such grounds? D. W. B.

—*South. Jour. of the Med. Sciences*, Aug., 1866.

